# Advances in Image-Guided Veterinary Surgery

**DOI:** 10.3390/ani15172530

**Published:** 2025-08-28

**Authors:** Antonello Bufalari, Andrea Corda, Domenico Caivano

**Affiliations:** 1 Department of Veterinary Medicine, University of Perugia, Via San Costanzo 4, 06126 Perugia, Italy; antonello.bufalari@unipg.it; 2 Department of Veterinary Medicine, University of Sassari, Via Vienna, 2, 07100 Sassari, Italy; andreacorda@uniss.it

## 1. Introduction

Image-guided surgery uses imaging systems as a preoperative and intraoperative guide for surgeons. The images obtained help surgeons navigate and perform the surgical procedure with greater accuracy and precision. Surgeons have a visual aid that allows them to see the patient’s anatomy in detail and monitor surgical instruments in relation to the target area to obtain a detailed and patient-specific anatomic description [1]. In human medicine, image-guided surgery is used in various surgical procedures, including neurosurgery, liver surgery and breast cancer surgery [2]. This approach enhances the safety and improves the accuracy of the surgery, reduces tissue trauma, helps to identify tumor margins and, finally, improves the outcome [2].

In the past few decades, medical imaging has been used as a preoperative and intraoperative guide for veterinary surgeons [3,4,5]. Imaging systems, such as X-rays, fluoroscopy, ultrasonography, computed tomography (CT) and magnetic resonance imaging (MRI) provide surgeons with a detailed and patient-specific anatomic description of the designated area before surgery [6,7]. Moreover, intraoperative imaging allows the surgeon to track surgical instruments in hybrid operating rooms in order to directly or indirectly guide the surgical procedures [6,7]. Imaging techniques can be used in open surgeries, minimally invasive surgeries or diagnostic/therapeutic procedures [3]. This multidisciplinary approach enhances the safety, improves the accuracy of the surgical procedure and reduces tissue trauma (especially using minimally invasive image-guided surgery or interventional procedures) [8,9,10].

The aim of this special edition is to collect the most significant and recent knowledge regarding the use of perioperative medical imaging techniques to support veterinary surgical procedures.

## 2. The Structure of This Special Issue

The Special Issue includes nine papers reporting the use of medical imaging techniques to help surgeons in cancer surgeries, cardiovascular procedures and orthopedic surgeries. [Gómez Ochoa et al., Contribution 1] demonstrates that percutaneous echo-guided radiofrequency ablation is an effective and safe procedure for treating large chemodectomas in dogs with clinical signs associated with this neoplasia. This technique offers a new and alternative approach in the clinical management of aortic body tumors, highlighting the utility of ultrasonography for the mini-invasive procedure. Laparoscopy is commonly used to assist cryptorchidectomy in horses: this imaging technique allows intra-abdominal inspection, facilitating access and manipulation of the testicle retained in the cavity. The handcrafted multiport device proposed by [Silva et al., Contribution 2] provides access to the abdominal cavity of horses undergoing laparoscopically assisted cryptorchidectomy, allowing intra-abdominal manipulation and removal of the cryptorchid testicles located in the inguinal region. Additionally, [Vidal-Negreira et al., Contribution 3] reported in their retrospective study the usefulness of the radiographic analysis in dogs with patellar ligament desmopathy following tibial plateau leveling osteotomy. Although other imaging techniques, such as ultrasonography or MRI, can offer greater detail, radiographic examination remains widely used due to its availability, objectivity and cost-effectiveness in clinical evaluations.

The review of [Caivano et al., Contribution 4] summarizes the current knowledge on the application of ultrasonography in the identification and removal of grass awns from various anatomic locations in dogs and cats. The scientific literature reviewed shows that ultrasonography is a valid diagnostic tool for visualization and guiding the removal of migrating grass awns in dogs and cats. Authors also highlighted that in complex cases, where the migration occurs in less accessible locations, ultrasonography should be considered as a part of a multidisciplinary approach with advanced diagnostic imaging modalities. [Abako et al., Contribution 5] characterize and compare the usefulness of imaging techniques available in veterinary medicine for the diagnosis and evaluation of lesions and injuries affecting the tarsal joint in dogs. Although several imaging techniques can be used in the diagnosis of lesions in the canine tarsal joint, authors suggest that only the simultaneous use of different imaging methods, in conjunction with clinical examination, allows for a full diagnosis.

Congenital cardiovascular defects, such as atrioventricular canal defect in a cat [Szaluś-Jordanow et al., Contribution 6] and segmental aplasia with azygos continuation of the caudal vena cava in a dog [Szatmári et al., Contribution 7], have been reported and diagnosed by ultrasonography and computed tomography. Pre- and postoperative ultrasonographic evaluations were conducted to monitor structural and functional changes in the heart and vessels [Szaluś-Jordanow et al., Contribution 6]. Additionally, fluoroscopy was fundamental for implantation of a self-expanding nitinol stent into the azygos vein at the level of the diaphragm, allowing the resolution of all clinical signs immediately after surgery [Szatmári et al., Contribution 7]. [Kymm et al., Contribution 8] reported usefulness of MRI and CT to reveal a mass in the fourth ventricle and caudal part of the brainstem, allowing the partial excision of the tumor using the telovelar approach. Finally, [Kim et al., Contribution 9] presented a novel application of indocyanine green-guided surgery and 18-Fluorodeoxyglucose Positron Emission Tomography/CT in a canine gastric tumor. This case highlights the novel application of indocyanine green as a near-infrared fluorescence agent for intraoperative identification of a gastric tumor and precise surgical margin assessment during gastrectomy.

These studies collectively illustrate the usefulness of the medical imaging techniques as support for the veterinary surgery.

## 3. Conclusions

This Special Issue highlights a variety of innovative contributions and reviews that report the use of perioperative medical imaging techniques in various veterinary surgical procedures. Medical imaging systems such as X-rays, fluoroscopy, ultrasonography, CT and PET allow for pre-operative planning, intraoperative guidance and minimally invasive interventions, leading to better outcomes and reduced tissue trauma. Additional studies are necessary to validate the effectiveness of these imaging technique in veterinary surgery and explore their use in other potential surgical procedures.
